# Prevalence, Severity, and Predictors of Poststroke Depression in a Prospective Cohort of Jordanian Patients

**DOI:** 10.1155/2022/6506326

**Published:** 2022-01-07

**Authors:** Majdi Al Qawasmeh, Belal Aldabbour, Amal Abuabada, Khalid Abdelrahman, Samah Elamassie, Mays Khweileh, Mohammad Zahran, Khalid El-Salem

**Affiliations:** ^1^Neurology Department, Faculty of Medicine, Jordan University of Science and Technology, Irbid, Jordan; ^2^Neuroscience Department, Faculty of Medicine, Islamic University of Gaza, P.O. Box 108, Gaza, State of Palestine; ^3^Psychiatry Department, Faculty of Medicine, Jordan University of Science and Technology, Irbid, Jordan; ^4^Health Services, United Nations Relief and Works Agency (UNRWA), Gaza, State of Palestine

## Abstract

Poststroke depression (PSD) is common and remains a significant risk factor for poor outcomes. This prospective study is aimed at assessing the prevalence, severity, and predictors of PSD among Jordanian stroke survivors. A total of 151 patients who were consequently admitted to a tertiary teaching hospital with ischemic or hemorrhagic strokes were enrolled. Participants were screened on admission for premorbid depression using the PHQ-9 questionnaire; then, screening for PSD was repeated one and three months after stroke using the same tool. Depression prevalence at each screening was reported, and logistic regression analysis was conducted to evaluate for significant predictors. PHQ-9 scores suggestive of depression were reported by 15%, 24.83%, and 17.39% of respondents on admission and after one and three months, respectively. Scores suggesting severe depression were reported by 0.71%, 2.13%, and 6.52% of respondents, respectively. Significant predictors of PSD were having chronic kidney disease, current smoking status, moderate or severe disability (mRS score) at stroke onset, and severe dependence (BI) after one month (**p** values 0.007, 0,002, 0.014, and 0.031, respectively). Patients with secondary and high school education levels were less likely to get depression compared with illiterate patients (**p** 0.042). This study showed that nearly one in four Jordanian stroke survivors experienced PSD after one month. In contrast, while the overall PSD prevalence declined towards the end of follow-up period, patients who remained depressed showed a tendency towards higher PSD severity.

## 1. Introduction

Stroke is a significant cause of morbidity and mortality globally [1]. It is the second leading noncommunicable cause of death in Jordan and a considerable source of complications and physical disability [2, 3]. Stroke survivors are liable to a multitude of physical, psychiatric, social, and functional impacts. Poststroke depression (PSD) is the most frequent and a very important neuropsychiatric complication. Stroke survivors who develop PSD are at a greater risk of poor functional outcomes, lower quality of life, increasing cognitive impairment, recurrent vascular events, and higher mortality than those without depression [4–6].

The DSM-V defines poststroke mood disorders as mood disorders due to stroke, with the specifiers of depressive features, major depressive-like episode, or mixed mood features [6]. Studies evaluating PSD have reported varying findings owing to heterogeneous settings, populations, and methodologies. Also, several studies suggested that PSD prevalence differs with the time interval between stroke and depression assessment [7]. Additionally, some neurological symptoms such as aphasia and cognitive impairment may conceal mood abnormalities, and there is a lack of consensus over the best screening tool for case-finding [7, 8]. Subsequently, the range of estimated PSD prevalence is wide [9, 10]. However, meta-analyses with large databases reported that approximately one-third of survivors developed PSD at any time point up to 5 years following stroke [11–13]. Surveys from the Middle East and North Africa (MENA) region also reported a wide prevalence of PSD (17-73%) [10], while previous surveys from Jordan reported a range from 25% to 76% [14–17]. Studies aiming to identify risk factors predisposing to PSD have also been inconsistent. The most frequently identified risk factors include the level of physical disability, stroke severity, and history of mental illness [7, 18].

Different instruments have been used to screen stroke survivors for PSD [19]. The Patient Health Questionnaire-9 (PHQ-9) is a self-administered tool that employs nine standard questions to score each of the nine DSM criteria as zero (not at all) to three (nearly every day) [20]. It has been well-validated in different settings as a brief screener for PSD [19, 21, 22]. A meta-analysis found that PHQ-9 had a summary sensitivity of 0.77 and a specificity of 0.94 (0.90–0.97) [23]. Cut-off PHQ-9 scores of 5, 10, 15, and 20 represent mild, moderate, moderately severe, and severe depression, respectively [20]. This study is aimed at determining the prevalence and severity of PSD at one and three months after stroke and at investigating PSD predictors among Jordanian stroke survivors.

## 2. Materials and Methods

### 2.1. Study Design, Setting, and Ethical Considerations

This prospective cohort study was conducted at King Abdullah University Hospital (KAUH), the teaching hospital of Jordan University of Science and Technology (JUST) Faculty of Medicine. With over 680 beds, KAUH is the largest tertiary and teaching hospital in the north of Jordan. It offers emergency, clinic, and inpatient health care services to residents from the four northern Jordanian governorates and receives referrals from other hospitals within these governorates as well. Ethical approval for this study was obtained from the Institutional Review Board Committee at JUST (IRB approval number 279-2019). Written consent was obtained from patients upon enrolment, and oral consent was obtained from patients who were unable to write appropriately. Participants with positive PSD screening were informed about their results and offered psychiatric consultations.

### 2.2. Study Population and Case Identification

The study period extended over ten months from June 2019 to March 2020. It included all patients above sixteen who were admitted to the Neurology Department at KAUH with the primary diagnosis of a first or recurrent ischemic or hemorrhagic stroke. The study excluded patients who were younger than 16, patients with subarachnoid, subdural, or epidural hemorrhage, patients in whom the final diagnosis was transient ischemic attacks (TIA) or diagnoses other than acute stroke, patients with severe aphasia or advanced dementia precluding meaningful communication, and patients who refused to participate. Patients with recurrent strokes during the study period were recruited once only, and screening and follow-up were limited to the period preceding the second event.

### 2.3. Key Measures

Stroke was defined according to the 2013 AHA definition for ischemic and hemorrhagic strokes [24]. Stroke symptoms were reported as motor, sensory, dysarthria, aphasia, and others. Stroke severity was assessed using NIHSS score, with stroke severity stratified as mild (NIHSS less than 6), moderate (NIHSS 6-15), severe (16–25), and very severe (more than 25) [2, 25]. Disability was assessed using the modified Rankin Score (mRS), and a favorable outcome was identified as having mRS score of 2 or less [2, 25]. Functional status was assessed with Barthel Index (BI) [25], with the level of dependence classified as total (scores 0-20), severe (21-60), moderate (61-90), slight (91-99), and complete independence (100) [26]. Depression screening was conducted using the validated Arabic version of the PHQ-9 scale [27, 28], with cut-off scores of 5, 10, 15, and 20 representing mild, moderate, moderately severe, and severe depression, respectively [20]. Hypertension, diabetes mellitus, and the other risk factors were defined according to standard guidelines, ascertained from previous notes or medical reports, or diagnosed by being on treatment for these conditions. Prolonged length of hospital stay (LOS) was defined as hospitalization lasting longer than the 75th percentile for the cohort [2].

### 2.4. Data Collection

Demographic data included age, gender, and education level. Each patient's final diagnosis, stroke severity on admission and discharge, stroke type, and presentation deficits were documented, as well as each patient's LOS and length of ICU stay. Stroke and PSD risk factors were ascertained, including hypertension, diabetes mellitus, heart failure, ischemic heart disease, cardiac arrhythmia, chronic kidney disease, previous ischemic or hemorrhagic stroke, smoking, and current or past history of psychiatric illness. Prestroke disability (mRS) and functional status (BI) were documented on admission and reassessed at discharge and after one month. As for depression screening, patients were screened on admission for premorbid depression, and screening was repeated via phone interviews conducted one and three months after discharge. In the last phone call, patients were asked if they had been to a psychiatrist or started on antidepressant medications after the stroke.

### 2.5. Statistical Analysis

Sample demographics and descriptive statistics were done first, reported as frequencies and proportions for categorical variables, and as mean (standard deviation “SD”) and median (interquartile range “IQR”) for continuous data. Chi-square and **t**-test were used to assess the relationship between categorical variables and means, respectively. Logistic regression analysis was done to investigate the significant predictors of depression one month after stroke. Independent variables included age, gender, level of dependence after one month, education status, level of disability on admission, length of ICU admission, active smoking status, prior mood symptoms, diabetes mellitus, hypertension, ischemic heart disease, atrial fibrillation, chronic kidney disease, and heart failure. All statistical analyses were run in the SPSS software. The significance level *α* was established at 0.05.

## 3. Results

**Figure 1:**
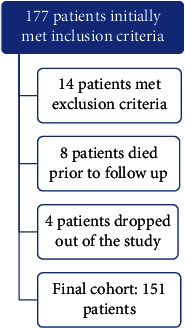
Study flowchart.

A total of 177 patients met the initial inclusion criteria, of which eight died within one month of the stroke, 11 were excluded due to severe stroke with persistent aphasia or impaired level of consciousness, two were excluded due to advanced dementia, and one due to language barriers precluding meaningful communication. Additionally, four patients dropped out of the study. Ultimately, 151 stroke survivors were included in the final analysis, of which 123 (81.45%) responded to all three screenings, while the remaining 28 (18.54%) missed either the first or the second follow-up screening after discharge.  Figure 1 demonstrates study flow chart.

### 3.1. Cohort Demographics, Risk Factor Profile, and Stroke Severity

Males constituted 66.23% of the cohort (100 patients) and females 33.77% (51 patients). The mean age of the final cohort was 62.74 years (SD 12.42). The mean age was 60.82 (SD 12.63) for males and 66.902 (SD 11.04) for females. The difference between the two means was statistically significant (*p* 0.004). A low level of education was notable among females as 34 (68%) were illiterate. On the other hand, 55 (55%) of male patients held a diploma, a university degree, or higher education, and 88.89% had any level of education. Most (135 patients, 89.40%) had at least one risk factor or comorbid medical condition before the stroke, with an overall mean of 2.377 (SD 1.43) comorbidities per patient. Hypertension was the most prevalent stroke risk factor (116, 76.82%), followed by diabetes mellitus (91, 60.26%) and previous stroke (48, 31.79%). Six (4.14%) patients reported a history of psychiatric comorbidity. Smoking was more prevalent among male patients as 51 (51%) were current smokers and 22 (22%) were ex-smokers, while 76.46% of female patients were lifetime nonsmokers.  Table 1 further illustrates these cohort demographics.

Most patients (142, 94%) suffered from an ischemic stroke, while 9 (6%) had an intracerebral hemorrhage (ICH). The most frequently reported neurological symptom was motor deficits (106 patients, 70.20%), followed by dysarthria (102 patients, 67.55%) and sensory deficits (50 patients, 33.11%). The mean admission NIHSS score was 5.79 (SD 5.08), with a mean NIHSS of 5.59 (SD 4.79) among male patients and 6.20 (SD 5.65) among females. Also, most patients had a favorable prestroke disability status as the mean prestroke mRS score for the entire cohort was 0.424 (SD 1.15), and 139 (92.05%) participants had a prestroke mRS score of two or less. The mean mRS score on discharge was 2.493 (SD 1.77), with 77 (51.33%) patients having a favorable discharge mRS score (2 or less). The number of patients with a favorable mRS increased to 97 (66.44%) one month after discharge. The mean discharge BI score was 68.66 (SD 33.84), and one month after discharge, it increased to 82.77 (SD 26.18). Mean LOS was 5.55 days (SD 5.57), and the median was four days (IQR 2-7). Prolonged LOS was noted in 32 (21.19%) participants. As for ICU admission, 46 (30.46%) of participants were admitted for at least one day, with a mean ICU stay of 4.11 (SD 3.59) days. Tables 2 and 3 further demonstrate these characteristics.

### 3.2. Depression Prevalence and Severity

As Table 4 shows, 140 patients were screened on admission for premorbid depression. Of those, 21 (15%) had positive screening. Scores suggestive of mild depression were reported by 15 patients (10.71%), while moderate depression was reported by four patients (2.85%). Scores suggesting moderately severe or severe depression were reported by one patient (0.71%) for each category. Females were significantly more likely to be affected by premorbid depression (23.91%) compared to males (10.63%) (Chi-square statistic = 4.2687, *p* = 0.039). Screening for depression one month after admission revealed that 35 patients (24.83%) reported any level of depression, representing an increase of 9.84% (Table 4). The percentage declined to 17.52% (24 patients) three months after admission, which was still higher than prestroke (admission) prevalence. Significantly, however, the severity of depression increased with time. Moderately severe and severe depression categories were reported by one patient each (0.71%) on admission. In comparison, six (4.26%) and seven (5.11%) patients reported PHQ-9 scores suggestive of moderately severe depression one and three months after admission, while severe depression was reported by three (2.13%) and nine (6.57%) patients, respectively. In terms of treatment, only seven patients took antidepressant medications in the three months following the stroke. Moreover, five patients only had seen a psychiatrist during the same interval.

Finally, we looked at the PHQ-9 questionnaire to assess which domains were most likely to be affected in each screening (Table 5). On admission, the two questions that were most likely to get higher scores were the second question (feeling down, depressed, or hopeless) and the third question (trouble falling or staying asleep or sleeping too much), and the mean score was highest for the third question. One month after stroke, the second, third, and fourth questions were most likely to be affected, with the highest mean reported for the second question followed by the third question. Three months after stroke, the first and the second questions were the most likely to receive a positive answer and recorded the highest means. Therefore, the most frequent complaint reported by patients at any interval was feeling down, depressed, or hopeless.

### 3.3. Predictors of Having PSD after One Month

Logistic regression analysis investigated the significant predictors of depression one month after stroke (Table 6). Among comorbidities, having chronic kidney disease increased the log odds of depression by 2.594 (*p* = 0.007). Active smoking status increased the log odds of depression by 2.187 (*p* = 0.002) versus nonsmokers and ex-smokers. Additionally, the binary logistic analysis revealed that patients with secondary and high school education levels were less likely to get depression than illiterate patients by log odd -2.372 (*p* = 0.042). We also found that patients with total and severe dependence (by BI) one month after stroke were more likely to be depressed by log odds of 4.523 and 1.690 (*p* 0.039 and *p* 0.031), respectively, compared with fully independent patients. Finally, patients with moderate or severe disability (mRS > 2) on admission were more likely to be depressed by log odds of 2.311 (*p* = 0.014) in comparison to patients with milder or no disability early after the stroke. The remaining variables were not statically significant.

## 4. Discussion

This study assessed the prevalence and severity of PSD among Jordanian stroke survivors one and three months after admission, comparing the results with prestroke prevalence in the same group. Approximately one in four patients in our cohort experienced PSD one month after the stroke, and although the prevalence decreased at three months after the event, those who remained depressed had a worse PSD severity. We also identified several predictors for having PSD in this population, including initial disability status, functional performance after one month, level of education, active smoking, and having chronic kidney disease.

Previous local studies have reported varying figures. For instance, the first study evaluating PSD in Jordan was conducted in 2001 [14]. The authors defined PSD according to the DSM-IV criteria for major depression and found that 25% of their cohort of 168 patients suffered from PSD three to four months after the event. They also reported that PSD was significantly more common among women survivors.

Another local study by Alghwiri in 2016 examined the relationship between PSD and physical well-being in a selected cohort of 61 Jordanian stroke survivors who were referred for physical therapy. It reported a PSD prevalence of 64% using the Beck Depression Inventory (PDI) questionnaire [16]. Similarly, Almhdawi et al. in 2020 evaluated 153 Jordanian stroke survivors with stroke chronicity of at least four months and who were receiving occupational therapy [17]. The investigators used the Depression Anxiety Stress Scale (DASS 21) and reported PSD in 74.5% of the cohort. However, both studies selected patients from physical or occupational therapy facilities, and participants had prominent physical disability and/or balance problems [16, 17]. Also, each of these studies had a different methodology and setting, and patients were screened at various intervals after stroke, which ultimately resulted in variable and generally noncomparable estimates of PSD prevalence.

Another cross-sectional study by Ayasrah et al. in 2017 evaluated PSD among 198 hospitalized patients with stroke in nine Jordanian hospitals using the Hospital Depression Subscale (HDS) of the Hospital Anxiety and Depression (HAD) scale [15]. The authors reported PSD in 51.6% of the sample. Yet again, different population characteristics, study settings, and screening tools may have predisposed for reporting such a high number. For instance, the mean age of patients was six years younger than our cohort, and 80.3% of the cohort were unable to perform activities of daily living without help (versus 40.39% of our cohort during admission). Also, patients were screened in a hospital setting, and the screening interval after the stroke was not standardized between patients. In addition, HDS has a lower positive predictive value (25-55%) for the used cut-off score than PHQ-9 (46-69%) [19, 29, 30], and the scale has one of the highest required literacy levels of the self-completed depression screening instruments [31].

On the other hand, three of the PSD risk factors identified by Ayasrah et al. correlated with our findings: having a lower level of education, being a smoker, and level of functional dependence after the stroke [15]. Physical disability and stroke severity also were significant predictors identified by Alghwiri in addition to cognitive impairment [16]. Therefore, our study's findings are consistent with previous literature and underline the considerable impact of stroke severity on the different outcome measures, including psychiatric complications [32].

Smoking was a significant predictor of PSD in both our study and the study by Ayasrah et al. [15]. It was also a significant predictor of PSD in other studies from our region and other regions [33, 34]. It has been suggested that this correlation may be due to vitamin D deficiency in smokers in addition to the dysphoric effects of nicotine abstinence [33, 34]. We suggest that it may also reflect feelings of guilt in stroke survivors with history of heavy smoking or in those who continue to smoke. This may be particularly relevant in Jordan, where the country is known to have one of the world's highest smoking rates [35] and where smoking has been linked to higher stroke severity after having a first ischemic stroke [2].

The natural history of poststroke depression is dynamic [36]. In this study, we found that the overall PSD prevalence was higher at the first month after stroke than prevalence at the third month, although severe PSD continued to increase with time. This finding likely reflects part of the natural history of PSD among Jordanian patients up to three months after stroke. In addition, other studies that have assessed stroke survivors more than once suggested that most patients had PSD soon after the event, and a majority had recovered in subsequent assessments [12]. Moreover, very few patients in our cohort saw a psychiatrist or took an antidepressant medication, which could have impacted their PSD severity on longitudinal follow-up.

Understanding the pathophysiology of PSD may decrease the uncertainty about its treatment and prevention strategies [7]. Nonetheless, the mechanisms underlying PSD are not well-defined owing to the complexity of its pathways and the heterogeneity of clinical studies [7, 18]. Possible contributing factors include lesion location [37], reductions in neurogenesis and/or angiogenesis [18, 38], immune dysfunction particularly in the limbic area [39], neurotransmitter dysfunction [40, 41], and hypothalamic-pituitary-adrenal (HPA) axis activation after stroke [42]. Moreover, it is believed that PSD is clinically underdiagnosed due to physician, patient, and caregiver under recognition [7, 18]. This highlights the necessity of increasing awareness about PSD symptoms among medical practitioners and the general population and demonstrates the need for a standard screening tool for PSD.

This study is not without limitations. It was conducted in a single center and excluded patients with advanced dementia or severe aphasia. Also, lasting effects of the stroke itself, such as residual neglect and cognitive impairment, may impact patient awareness about their symptoms. Additionally, follow-up was limited to three months after the stroke. Finally, although PHQ-9 is a recognized screening tool for PSD, the gold standard for diagnosis remains through clinician-administered structured clinical interviews.

## 5. Conclusions

Approximately one in four Jordanian stroke survivors in our cohort experienced PSD one month after the stroke. Although prevalence at three months was lower, the severity of depression increased towards the end of follow-up. Significant PSD predictors included the initial disability status, functional performance after one month, level of education, active smoking, and having chronic kidney disease. This study demonstrates the need for better awareness about PSD among medical practitioners and the general population and underscores the global need for a standard screening tool for PSD.

## Figures and Tables

**Table 1:** Cohort demographics and risk factor profile.

Variables	Categories	*N* 151	%	Male (%) *N* 100	Female (%) *N* 51
Gender	Male	100	66.23		
	Female	51	33.77		
Age	50 or less	25	16.56	19 (19.00)	6 (11.76)
	Above 50	126	83.44	81 (81.00)	45 (88.24)

Education level^∗^	Illiterate	45	30.21	11 (11.11)	34 (68.00)
	Primary school	20	13.40	15 (15.15)	5 (10.00)
	Secondary and high school	24	16.11	18 (18.18)	6 (12.00)
	Graduated and diploma	51	34.23	46 (46.46)	5 (10.00)
	Higher education	9	6.04	9 (9.09)	0 (0.00)

Smoking status	Not a smoker	66	43.71	27 (27.00)	39 (76.47)
	Smoker	58	38.41	51 (51.00)	7 (13.73)
	Ex-smoker	27	17.88	22 (22.00)	5 (9.80)
	Not a smoker and ex-smoker	93	61.59%	49 (49.00)	44 (86.27)

Comorbidities	Hypertension	116	76.82	71 (71.00)	45 (88.24)
	Diabetes	91	60.26	58 (58.00)	33 (64.71)
	Previous stroke	48	31.79	29 (29.00)	19 (37.25)
	Ischemic heart disease	46	30.67	27 (27.00)	19 (37.30)
	Heart failure	16	10.60	8 (8.00)	8 (15.69)
	Atrial fibrillation	14	9.33	6 (6.10)	8 (15.69)
	Chronic kidney disease	14	9.27	9 (9.00)	5 (9.80)
	Psychiatric comorbidity	6	4.14	4 (4.12)	2 (4.17)
	Having other diseases	32	21.19	18 (18.00)	14 (27.45)

Total number of comorbidities	No comorbidities	16	10.60	15 (15.00)	1 (1.96)
	One comorbidity	23	15.23	16 (16.00)	7 (13.73)
	Two comorbidities	46	30.46	29 (29.00)	17 (33.33)
	Three comorbidities	35	23.18	26 (26.00)	9 (17.65)
	Four comorbidities	20	13.25	9 (9.00)	11 (21.57)
	Five comorbidities	7	4.64	4 (4.00)	3 (5.88)
	Six comorbidities	4	2.65	1 (1.00)	3 (5.88)

^∗^Variability in totals is due to a missing value for one patient.

**Table 2:** Stroke type and severity.

Variables	Categories	*N* 151	%	Male (%) *N* 100	Female (%) *N* 51
Type of stroke	Ischemic	142	94.00	95 (95.00)	47 (92.16)
	Hemorrhagic	9	6.00	5 (5.00)	4 (7.84)

Length of hospital stay (LOS)	1-2 days	38	25.17	24 (24.00)	14 (27.45)
	3-4 days	51	33.77	35 (35.00)	16 (31.37)
	5-7 days	30	19.87	20 (20.00)	10 (19.61)
	7-42 days	32	21.19	21 (21.00)	11 (21.57)

No ICU admission		105	70.47	72 (73.47)	33 (64.71)

Patients admitted to ICU	1-4	27	61.36	17 (65.38)	10 (55.56)
	More than 4	17	38.64	9 (34.62)	8 (44.44)

Admission NIHSS scores	Mild	84	56.00	57 (57.00)	27 (54.00)
	Moderate	57	38.00	38 (38.00)	19 (38.00)
	Severe	8	5.33	5 (5.00)	3 (6.00)
	Very severe	1	0.67	0 (0.00)	1 (2.00)

Presenting symptoms	Motor deficit	106	70.20	73 (73.00)	33 (64.71)
	Dysarthria	102	67.55	69 (69.00)	33 (64.71)
	Sensory deficit	50	33.11	32 (32.00)	18 (35.29)
	Dysphasia	22	14.67	13 (13.13)	9 (17.65)
	Unsteadiness	8	5.30	6 (6.00)	2 (3.92)
	Vision problem	7	4.63	6 (6.00)	1 (1.96)
	Seizure	4	2.65	3 (3.00)	1 (1.96)
	Vertigo	3	1.99	2 (2.00)	1 (1.96)
	Dysphagia	3	1.98	1 (1.00)	2 (3.92)
	Ataxia	1	0.66	1 (1.00)	0 (0.00)

**Table 3:** Disability and functional status.

Variables	Before stroke		At discharge		At one month	
	*N*	%	*N*	%	*N*	%
Modified Rankin score (dichotomous representation)						
0-2	139	92.05	77	51.33	97	66.44
3-5	12	7.95	73	48.67	49	33.56

Modified Rankin score (detailed)						
0 no symptoms	127	84.11	20	13.33	44	29.93
1 no significant disability	8	5.30	46	30.67	43	28.57
2 slight disability	4	2.65	11	7.33	11	7.48
3 moderate disability	4	2.65	7	4.67	15	10.20
4 moderately severe disability	4	2.65	45	30.00	27	18.73
5 severe disability	4	2.65	21	14.00	7	4.76
Mean (SD)	0.424 (1.15)		2.493 (1.77)		1.78 (1.78)	

Barthel Index						
Total dependence (0-20)	—	—	19	12.58	6	3.97
Severe dependence (21-60)	—	—	42	27.81	22	14.57
Moderate dependence (61-90)	—	—	22	14.57	32	21.19
Slight dependence (91-99)	—	—	10	6.62	11	7.28
Full independence (100)	—	—	58	38.41	80	52.98
Mean (SD)	—		68.66 (33.84)		82.77 (26.18)	

**Table 4:** Prevalence of depression.

Factors	PHQ-9 score (depression severity)				
	0-4 (no depression) *N* (%)	5-9 (mild depression) *N* (%)	10-14 (moderate depression) *N* (%)	15-19 (moderately severe) depression *N* (%)	20-27 (severe depression) *N* (%)
Depression screening on admission					
Total sample (*N* 140)^∗^	119 (85.00%)	15 (10.71%)	4 (2.86%)	1 (0.71%)	1 (0.71%)
Males (*N* 94)	84 (89.36%)	8 (8.51%)	1 (1.06%)	0 (0.00%)	1 (1.06%)
Females (*N* 46)	35 (76.09%)	7 (15.22%)	3 (6.52%)	1 (2.17%)	0 (0.00%)
Age above 50 (*N* 115)	97 (84.35%)	14 (12.17%)	3 (2.61%)	1 (0.87%)	0 (0.00%)
Age 50 or less (*N* 25)	22 (88.00%)	1 (4.00%)	1 (4.00%)	0 (0.00%)	1 (4.00%)
Illiterate (*N* 40)^∗∗^	32 (80.00%)	4 (10.00%)	3 (7.50%)	1 (2.50%)	0 (0.00%)
Primary education (*N* 16)	13 (76.47%)	2 (11.76%)	1 (5.88%)	0 (0.00%)	1 (5.88%)
Secondary or high school (*N* 23)	20 (86.96%)	3 (13.04%)	0 (0.00%)	0 (0.00%)	0 (0.00%)
Graduate (*N* 49)	45 (91.84%)	4 (8.16%)	0 (0.00%)	0 (0.00%)	0 (0.00%)
Postgraduate (*N* 9)	8 (88.89%)	1 (11.11%)	0 (0.00%)	0 (0.00%)	0 (0.00%)
Depression screening one month after stroke					
Total sample (*N* 141)	106 (75.18%)	13 (9.22%)	13 (9.22%)	6 (4.26%)	3 (2.13%)
Male (*N* 93)	72 (77.42%)	10 (10.75%)	6 (6.45%)	3 (3.23%)	2 (2.15%)
Female (*N* 48)	34 (70.83)	3 (6.25%)	7 (14.58%)	3 (6.25%)	1 (2.08%)
Age above 50 (*N* 117)	86 (73.50%)	11 (9.40%)	12 (10.26%)	6 (5.13%)	2 (1.71%)
Age 50 or less (*N* 24)	20 (83.33%)	2 (8.33%)	1 (4.17%)	0 (0.00%)	1 (4.17%)
Illiterate (*N* 41)	29 (70.73%)	4 (9.76%)	6 (14.63%)	1 (2.44%)	1 (2.44%)
Primary education (*N* 17)	10 (58.82%)	3 (17.65%)	2 (11.76%)	1 (5.88%)	1 (5.88%)
Secondary or high school (*N* 23)	20 (86.96%)	0 (0.00)	3 (13.04%)	0 (0.00)	0 (0.00)
Graduate (*N* 49)	38 (77.55%)	5 (10.20%)	2 (4.08%)	3 (6.12%)	1 (2.04%)
Postgraduate	8 (88.9%)	1 (11.11%)	0 (0.00%)	0 (0.00%)	0 (0.00%)
Depression screening three months after stroke					
Total sample (*N* 137)	113 (82.48%)	2 (1.46%)	6 (4.38%)	7 (5.11%)	9 (6.57%)
Male (*N* 92)	79 (85.87%)	0 (0.00%)	3 (3.26%)	5 (5.43%)	5 (5.43%)
Female (*N* 46)	35 (76.09%)	2 (4.34%)	3 (6.52%)	2 (4.34%)	4 (8.70%)
Age above 50 (*N* 116)	96 (82.76%)	2 (1.72%)	4 (3.45%)	6 (5.17%)	8 (6.90%)
Age 50 or less (*N* 22)	18 (81.82%)	0 (0.00%)	2 (9.09%)	1 (4.54%)	1 (4.54%)
Illiterate (*N* 42)	35 (83.33%)	1 (2.38%)	1 (2.38%)	2 (4.76%)	3 (7.14%)
Primary education (*N* 18)	13 (72.22%)	1 (5.56%)	2 (11.11%)	0 (0.00%)	2 (11.11%)
Secondary or high school (*N* 22)	18 (81.82%)	0 (0.00%)	2 (9.09%)	0 (0.00%)	2 (9.09%)
Graduate (*N* 47)	40 (85.10%)	0 (0.00%)	1 (2.13%)	5 (10.64%)	1 (2.13%)
Postgraduate (*N* 7)	7 (100.00%)	0 (0.00%)	0 (0.00%)	0 (0.00%)	0 (0.00%)

^∗^11 patients were not screened on admission due to severe stroke symptoms (e.g., dysarthria or aphasia). ^∗∗^Education data was missing for three patients.

**Table 5:** The most affected domain at different screening periods.

Screening time	On admission		After one month		After three months	
Number and percentage of participants who answered yes and the mean score for all respondents	*N* = 140 (%)	Mean (SD)	*N* = 141 (%)	Mean (SD)	*N* = 26 (%)^∗^	Mean (SD)
Patient Health Questionnaire						
Q1 Little interest or pleasure in doing things	25 (17.85)	0.27 (0.655)	36 (25.53)	0.51 (0.956)	25 (96.15)	2.38 (0.852)
Q2 Feeling down, depressed, or hopeless	33 (23.57)	0.33 (0.662)	41 (29.08)	0.59 (1.011)	25 (96.15)	2.23 (0.951)
Q3 Trouble falling or staying asleep or sleeping too much	31 (22.14)	0.35 (0.719)	42 (29.79)	0.58 (0.990)	21 (80.77)	1.96 (1.148)
Q4 Feeling tired or having little energy	23 (16.36)	0.26 (0.650)	42 (29.79)	0.52 (0.893)	24 (92.31)	2.08 (1.017)
Q5 Poor appetite or overeating	20 (14.28)	0.24 (0.645)	27 (19.15)	0.37 (0.834)	16 (61.54)	1.27 (1.185)
Q6 Feeling bad about yourself—or that you are a failure or have let yourself or your family down	8 (5.71)	0.11 (0.475)	11 (7.80)	0.15 (0.574)	23 (88.46)	1.54 (0.905)
Q7 Trouble concentrating on things, such as reading the newspaper or watching television	26 (18.75)	0.31 (0.740)	33 (23.40)	0.39 (0.792)	21 (80.77)	1.62 (1.134)
Q8 Moving or speaking so slowly that other people could have noticed? Or so fidgety or restless that you have been moving a lot more than usual	6 (4.28)	0.07 (0.372)	12 (8.51)	0.14 (0.503)	20 (76.92)	1.81 (1.167)
Q9 Thoughts that you would be better off dead, or thoughts of hurting yourself in some way	13 (9.29)	0.14 (0.4383)	15 (10.64)	0.20 (0.659)	18 (69.23)	1.19 (1.021)

^∗^The number represents patients who are depressed, who have been on an antidepressant medication, or both.

**Table 6:** Predictors of depression after one month.

Predictors of depression in the first month poststroke				
Factors	*B*	Wald	Sig.	Exp (*B*)
Gender	0.475	0.365	0.546	1.608
Age (reference less than 50)	-0.361	0.197	0.657	0.697
Barthel Index one month after the event				
Total dependency (0-20)	4.523	4.246	0.039	92.145
Severe dependency (21-60)	1.690	4.632	0.031	5.418
Moderate dependency (61-90)	0.377	0.239	0.625	1.458
Slight dependency (91-99)	1.139	1.527	0.217	3.123
Education level				
Primary school	0.483	0.326	0.568	1.621
Secondary and high school	-2.372	4.122	0.042	0.093
Graduated and diploma	-0.251	0.106	0.745	0.778
Postgraduate	-2.255	2.032	0.154	0.105
Modified ranking score on admission (reference 0-2)	2.311	6.029	0.014	10.087
Comorbidities and risk factors				
Diabetes	-0.195	0.112	0.738	0.823
Hypertension	0.699	0.800	0.371	2.011
Ischemic heart disease	-0.873	1.922	0.166	0.418
Atrial fibrillation	-1.442	1.072	0.301	0.237
Chronic kidney disease	2.594	7.249	0.007	13.384
Heart failure	0.454	0.180	0.672	1.575
Number of days the patients admitted to ICU	0.174	1.784	0.182	1.190
Smoking (reference not smoker and ex-smoker)	2.187	9.497	0.002	8.912
Prior mood symptoms	-1.951	2.037	0.154	0.142

## Data Availability

The data is available from Belal Aldabbour upon reasonable request.
